# Multistability in a star network of Kuramoto-type oscillators with synaptic plasticity

**DOI:** 10.1038/s41598-021-89198-0

**Published:** 2021-05-10

**Authors:** Irmantas Ratas, Kestutis Pyragas, Peter A. Tass

**Affiliations:** 1 Center for Physical Sciences and Technology, 10257 Vilnius, Lithuania; 2 Department of Neurosurgery, School of Medicine, Stanford University, Stanford, CA 94305 USA

**Keywords:** Statistical physics, thermodynamics and nonlinear dynamics, Computational science

## Abstract

We analyze multistability in a star-type network of phase oscillators with coupling weights governed by phase-difference-dependent plasticity. It is shown that a network with *N* leaves can evolve into $2^N$ various asymptotic states, characterized by different values of the coupling strength between the hub and the leaves. Starting from the simple case of two coupled oscillators, we develop an analytical approach based on two small parameters $\varepsilon$ and $\mu$, where $\varepsilon$ is the ratio of the time scales of the phase variables and synaptic weights, and $\mu$ defines the sharpness of the plasticity boundary function. The limit $\mu \rightarrow 0$ corresponds to a hard boundary. The analytical results obtained on the model of two oscillators are generalized for multi-leaf star networks. Multistability with $2^N$ various asymptotic states is numerically demonstrated for one-, two-, three- and nine-leaf star-type networks.

Real-world networks often consist of connected active elements and change their structure over time. Typically, links between network elements are reorganized in response to changes in the states of the elements. Examples of such behavior can be found in chemical, biological and social systems^1^. One of the most intriguing systems that can reconfigure its connections is a neural network.

Spike-timing-dependent plasticity (STDP) refers to a concept of neural network formation in which the relative timing between the presynaptic inputs and postsynaptic firing dictates the direction of the synaptic strength modulation^2,3^. Presynaptic firing followed by a postsynaptic spike induces potentiation (the synaptic weight increases), while postsynaptic firing occurring before presynaptic firing leads to depression (the synaptic weight decreases)^4,5^. It is believed that such activity-dependent changes in synaptic transmission provide a neural basis for the realization of higher brain functions, such as learning and memory.

STDP models prescribe the functional relation between the synaptic modification and the time difference between a pair of pre- and postsynaptic action potentials. Effective modification of the synaptic weight takes place only when the spike-timing difference is within a certain time interval (learning window). Typically, asymmetrical learning windows are observed with a change in the sign of the spike-timing difference^6–8^. Numerical simulations of STDP-driven neuronal networks show that the asymmetry between potentiation and depression leads to multistability characterized by different levels of synchrony^9–11^. The effect of STDP on synaptic weights depends not only on the STDP learning function, but also on how the boundaries are implemented in the model to maintain synaptic weights in the allowable range. Various types of boundaries were considered, including soft (multiplicative) boundaries^12,13^, hard (additive) boundaries^14,15^, as well as interpolation between soft and hard boundaries^16^.

Modeling plastic neural networks is a complex numerical task for two reasons: (i) neural dynamics and adaptation of synaptic weights usually occur at different time scales; (ii) the number of dynamic variables associated with the slow adaptation of synaptic weights increases quadratically with the size of the network. Because of these difficulties, various simplifications are used. One way is to implement networks with simplified neural models. Most popular in numerical analysis are plastic networks of leaky integrate-and-fire neurons^17–19^, which enable event-driven simulations^20^. Other type of models used in plastic networks are phase oscillators^9,21^ (Kuramoto-type networks^22,23^). These models often implement simplified STDP rules^10,11,24–28^.

In this paper, we consider Kuramoto-type networks with coupling weights governed by a phase-difference-dependent plasticity (PDDP) rule, first introduced in Ref.^10^. The PDDP rule is built similarly to the STDP rule, but instead of the time difference, the phase difference of the oscillators is used, and the instantaneous change in synaptic weights is replaced by their continuous change in time. The relationship between PDDP and STDP, obtained by averaging STDP over time, is discussed in Ref.^27^. PDDP has an advantage over STDP in that it is more amenable to analytical analysis. We investigate the PDDP effect on the formation of a star-type network. In the star network, there is a center node (hub), and each of the other nodes (leaves) is connected only to this center but not between each other. The star network can be considered as an essential building block in real neural networks^29–31^; it is the simplest network model that captures the sparse, clustering, small-world and other important properties of many real-world networks^32–34^. An advantage of a star network over more complex networks is that the number of dynamic variables associated with synaptic weights increases linearly, rather than quadratically, with the size of the network. Non-plastic star networks of phase oscillators were considered in Refs.^35–37^. Here we analyze the multistable dynamics of such networks caused by PDDP.

## Model

We consider a star network with $N+1$ nodes described by Kuramoto-type phase oscillators:

1a $$\begin{aligned} \dot{\theta }_{0}= & {} \omega _{0}+\sum _{k=1}^{N}A_{k}\sin \left( \theta _{k}-\theta _{0}\right) , \end{aligned}$$

1b $$\begin{aligned} \dot{\theta }_{j}= & {} \omega _{j}+B_{j}\sin \left( \theta _{0}-\theta _{j}\right) , \quad j=1,\ldots , N. \end{aligned}$$

Here $\theta _0$ is the phase of the central node (hub) and $\omega _0$ is its natural frequency (spiking rate). The variables $\theta _j$ and the parameters $\omega _j$ for $j=1,\ldots , N$ are the phases and the natural frequencies of the leaves, respectively. The hub is forced by all leaves, and each leaf is forced by the hub. The parameter $A_{k}$ indicates the synaptic weight of the directional link from the *k*th leaf to the hub, and $B_{j}$ is the synaptic weight of the directional link from the hub to the *j*th leaf. We modify the synaptic weights $A_j$ and $B_j$ in dependence on the phase difference

2 $$\begin{aligned} \varphi _j=\theta _0-\theta _j, \quad j=1,\ldots , N \end{aligned}$$

between the hub and the *j*th leaf. For PDDP, we adopt STDP rules typical of excitatory synapses^6,15^. When the leaf phase is ahead of the hub phase ($\varphi _j<0$), the synaptic weight $A_j$ increases, and the synaptic weight $B_j$ decreases. In the opposite case, $\varphi _j>0$, the weight $A_j$ decreases, and the weight $B_j$ increases. More specifically, we modify synaptic weights using the time-continuous form of the PDDP rule introduced in Ref.^10^:

3a $$\begin{aligned} \dot{A}_j= & {} \varepsilon {\left\{ \begin{array}{ll} F(\alpha -A_j)\exp \left( \varphi _j/\tau _{+}\right) , &{} \varphi _j \in \left[ -\pi , 0 \right) ,\\ -F(A_j)\exp \left( -\varphi _j/\tau _{-}\right) , &{} \varphi _j \in \left[ 0,\pi \right) , \end{array}\right. } \end{aligned}$$

3b $$\begin{aligned} \dot{B}_j= & {} \varepsilon {\left\{ \begin{array}{ll} -F(B_j)\exp \left( \varphi _j/\tau _{-}\right) , &{} \varphi _j\in \left[ -\pi , 0 \right) ,\\ F(\alpha -B_j)\exp \left( -\varphi _j/\tau _{+}\right) , &{} \varphi _j \in \left[ 0,\pi \right) . \end{array}\right. } \end{aligned}$$

Here $\varepsilon \ll 1$ is a small but not vanishing parameter that takes into account the slow change in synaptic weights relative to the fast phase dynamics. The learning windows over which post- (pre-) synaptic spikes will cause synaptic potentiation (depression) are indicated as $\tau _+$ ($\tau _-$). Following experimental evidences^6–8^, we assume $\tau _->\tau _+$. In numerical experiments below, we choose^10^: $\tau _-=0.3$ and $\tau _+=0.15$. The function *F*(*x*) is introduced to satisfy the boundary conditions, that is, to keep the synapses from achieving unrealistically large values or becoming inhibitory, namely $0\le A_j \le \alpha$ and $0\le B_j \le \alpha$. Various types of boundary functions are considered in the literature. A soft boundary is associated with the linear function $F(x) = x$ as in Refs.^12,13^, and a hard boundary is determined by the Heaviside step function *H*(*x*), $F(x)=H(x)$ as in Refs.^14,15^. Interpolation between soft and hard boundaries can be achieved by a power function^16^

4 $$\begin{aligned} F(x) = x^\mu \end{aligned}$$

with a sharpness parameter $\mu$ varying in the interval $0<\mu \le 1$. For $\mu =1$ this function corresponds to the soft boundary, and for $\mu \rightarrow 0$ it is the case of the hard boundary. In this paper, to analyze the transition to a hard boundary, we will mainly use the sigmoid function

5 $$\begin{aligned} F(x) = \tanh (x/\mu ). \end{aligned}$$

Like function (4), this function vanishes at $x=0$ and monotonically increases with an increase of *x*. The parameter $\mu$ determines the sharpness of the transition from zero to one: $F(x)\approx x/\mu$ for $x \ll \mu$ and $F(x)\approx 1$ for $x\gg \mu$. The hard boundary corresponds to the limit $\mu \rightarrow 0$. Note that the function (5) is analytic at $x=0$ for any $\mu$, while the function (4) is non-analytic at $x=0$ for $0<\mu <1$.

From the Eq. (1) we can derive equations for phase differences (2):

6 $$\begin{aligned} \dot{\varphi }_j=\omega _0-\omega _j-B_j \sin (\varphi _j)-\sum _{k=1}^{N}A_{k}\sin (\varphi _k). \end{aligned}$$

Thus, the dynamics of a star-type plastic network with *N* leaves is determined by a closed system of 3*N* differential Eqs. (3) and (6). In this system, 2*N* Eq. (3) describe the slow adaptation of synaptic weights, and *N* Eq. (6) determine the fast variation of phase differences.

## Methods

**Two oscillators under PDDP.** The case of two oscillators allows for analytical treatment. The analytical results obtained in this section are used to predict the asymptotic behavior of multi-leaf star networks. In the case of two oscillators, $N=1$ and the system’s state is characterized by three dynamical variables $A_1$, $B_1$ and $\varphi _1$. Since the variable $\varphi _1$ is fast with respect to the variables $A_1$ and $B_1$, we can eliminate it from the system’s equations. The slow variables $A_1$, $B_1$ are governed by the Eq. (3) with $j=1$, and the Eq. (6) for the fast phase variable $\varphi _1$ reads

7 $$\begin{aligned} \dot{\varphi }_1=\Delta -K \sin (\varphi _1), \end{aligned}$$

where

8 $$\begin{aligned} K=A_1+B_1 \end{aligned}$$

is the sum of the synaptic weights, and

9 $$\begin{aligned} \Delta =\omega _0-\omega _1 \end{aligned}$$

is the frequency detuning between the oscillators. Without loss of generality, we assume $\omega _0>\omega _1$, so the parameter $\Delta$ is positive. Due to the small parameter $\varepsilon$, we can neglect the variations of the slow variables^38^ $A_1$ and $B_1$ on the fast time scale of the variable $\varphi _1$. For the constant *K*, the solution of Eq. (7) depends on the ratio $K/\Delta$. When this ratio is greater than one, the phases of the oscillators are locked, and when it is less than one the oscillators are not synchronized. Below we consider these two cases separately.

(i) Case $K>\Delta$. Under this condition, the Eq. (7) has two fixed points, one of which is stable:

10 $$\begin{aligned} \varphi _1^{*}(K)=\arcsin \left( \Delta /K\right) . \end{aligned}$$

It determines the phase difference of the locked oscillators in dependence of the variable *K*. Substituting this phase difference in Eq. (3) and taking into account that $\varphi _1^{*}(K) \in (0, \pi )$, we obtain the following approximate system to describe the slow dynamics of the synaptic weights $A_1$ and $B_1$:

11a $$\begin{aligned} \dot{A}_1= & {} -\varepsilon F(A_{1})\exp \left[ -\varphi _1^{*}(K)/\tau _{-}\right] ,\end{aligned}$$

11b $$\begin{aligned} \dot{B}_1= & {} \varepsilon F(\alpha -B_{1})\exp \left[ -\varphi _1^{*}(K)/\tau _{+}\right] . \end{aligned}$$

The condition $F(0)=0$ implies that this system has a fixed point

12 $$\begin{aligned} (A_1^{*}, B_1^{*})=(0,\alpha ). \end{aligned}$$

Linear stability analysis of the system (11) shows that the fixed point (12) is stable due to $F'(0)>0$. This fixed point describes an asymptotic synchronized state of the system in which the oscillators become unidirectionally coupled. The directional link from the slower oscillator (with the natural frequency $\omega _1$) to the faster oscillator (with the natural frequency $\omega _0$) becomes disconnected, while the synapse associated with the directional link from a faster oscillator to the slower oscillator, reaches the maximum value $\alpha$. The instantaneous frequencies of the synchronized oscillators become equal to the natural frequency of the faster oscillator: $\dot{\theta }_1=\dot{\theta }_0=\omega _0$. Since Eq. (11) are valid for $A_1+B_1>\Delta$, the necessary condition for the existence of the synchronized state (12) is

13 $$\begin{aligned} \alpha >\Delta . \end{aligned}$$

It is important to note that the above synchronized state also exists in the original (non-reduced) system of Eqs. (3) and (7) without the assumption that the parameter $\varepsilon$ is small. Indeed, in the three-dimensional state space $(A_1, B_1, \varphi _1)$ of this system there is always a stable fixed point $(A_1^{*}, B_1^{*}, \varphi _1^*)=(0,\alpha , \arcsin (\Delta /\alpha ))$ if the condition (13) is met. Thus, the synchronized state with disabled synaptic connection $A_1 = 0$ and maximum synaptic connection $B_1 = \alpha$ is exact for any values of the parameters $\varepsilon$ and $\mu$.

(ii) Case $K<\Delta$. Under this condition, the oscillators are not synchronized and the phase difference $\varphi _1$ shows fast oscillations. Again, we neglect the slow variations of $A_1$ and $B_1$ on the fast time scale. For constant *K*, the oscillation period can be obtained from the Eq. (7) in analytical form:

14 $$\begin{aligned} T(K) = \int _0^{2\pi } \frac{d\varphi _1}{\Delta -K\sin (\varphi _1)} = \frac{ 2\pi }{\sqrt{\Delta ^2 -K^2}}. \end{aligned}$$

Now an approximate system of equations for the slow variables $A_1$ and $B_1$ can be obtained by averaging the Eq. (3) over the period of fast oscillations^39^:

15a $$\begin{aligned} \dot{A}_{1}= & {} \frac{\varepsilon }{T(K)}\biggl [F(\alpha -A_{1})\int _{-\pi }^{0}G_{+}(K,\varphi _1)\,{\text{d}}\varphi _1 -F(A_{1})\int _{0}^{\pi }G_{-}(-K,-\varphi _1)\,{\text{d}}\varphi _1 \biggr ],\end{aligned}$$

15b $$\begin{aligned} \dot{B}_{1}= & {} \frac{\varepsilon }{T(K)}\biggl [ F(\alpha -B_{1})\int _{0}^{\pi }G_{+}(-K,-\varphi _1)\,{\text{d}}\varphi _1 -F(B_{1})\int _{-\pi }^{0}G_{-}(K,\varphi _1)\text {d}\varphi _1\biggr ], \end{aligned}$$

where

16 $$\begin{aligned} G_{\pm }(K,\varphi _1)= \frac{\exp \left( \varphi _1/\tau _{\pm }\right) }{\left[ \Delta -K\sin \left( \varphi _1\right) \right] }. \end{aligned}$$

**Figure 1:**
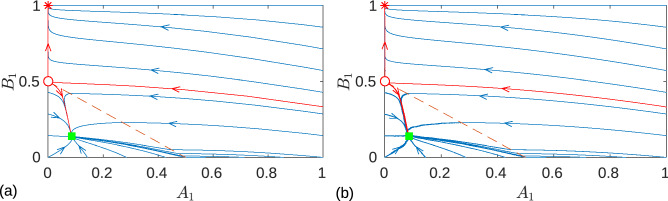
Comparisson of (**a**) the phase portrait in the plane of slow variables ($A_1, B_1$) obtained from the reduced system of Eqs. (11) and (15) with (**b**) the projection of the solution of the original system of Eqs. (3) and (7) to the same plane ($A_1, B_1$). The red dashed line $A_1+B_1=\Delta$ divides the areas of synchronized ($A_1+B_1>\Delta$) and unsynchronized ($A_1+B_1<\Delta$) motion of the oscillators. The red star and green square indicate the stable fixed points of the synchronized and desynchronized system, respectively. The red circle marks a saddle point, and red trajectories represent its separatrices. The stable separatrix separates the basins of attraction of the two stable fixed points. The red circle and green square represent true fixed points in panel (**a**) only, while in panel (**b**) they can only be roughly interpreted as fixed points (see main text for details). The results are presented for the sigmoid boundary function (5) whith the parameter $\mu =0.2$. Other parameters are: $\omega _0=1$, $\omega _1=0.5$, $\alpha = 1$, $\tau _+ = 0.15$, $\tau _-=0.3$, and $\varepsilon = 0.01$.

To summarize, the Eqs. (11) and (15) together with expressions (8), (10), (14) and (16) constitute a complete system of reduced differential equations for describing the dynamics of the system in the entire plane ($A_1, B_1$) of slow variables. Unlike the original system of three differential Eqs. (3) and (7), here the fast variable $\varphi _1$ is eliminated. The line $A_1+B_1=\Delta$ in the plane of slow variables divides the areas of synchronized and unsynchronized motion of oscillators (see the red dashed line in Fig. 1a). The region $A_1+B_1>\Delta$ corresponds to synchronized motion and is described by Eq. (11), while the region $A_1+B_1<\Delta$ represents unsynchronized motion and is defined by Eq. (15).

To justify the validity of the reduced system Eqs. (11) and (15), in Fig. 1 we compare the phase portrait in the plane of variables ($A_1, B_1$) obtained from the reduced system (Fig. 1a) with the projection of the solution of the original system of three differential Eqs. (3) and (7) to the same plane ($A_1, B_1$) (Fig. 1b). For $\varepsilon =0.01$, we see excellent agreement between these two results. The red star is the fixed point (12) described above, which corresponds to the synchronized state of oscillators with a maximum synaptic weight $B_1$ and a vanishing synaptic weight $A_1$. We see that there is another stable fixed point located in the unsynchronized region below the red dashed line marked by green square. This means that the system is bistable with coexisting synchronized and unsynchronized steady states. The basins of attraction of stable fixed points are separated by the stable separatrix of the saddle point, which is marked with a red circle. The saddle is in the unsynchronized region, just below the red dashed line. Strictly speaking, the term “fixed point” in the unsynchronized region is correct only within the framework of the reduced system of Eq. (15). In the original system of Eqs. (3) and (7), the variables $A_1$ and $B_1$ show high-frequency oscillations around the usynchronized steady state with a small amplitude proportional to $\varepsilon$. The period of these oscillations is determined by the Eq. (14). At $\varepsilon \rightarrow 0$ the amplitude of these oscillations tends to zero. Numerical simulation of the original system of Eqs. (3) and (7) showed that convergence to asymptotic states does not depend on the initial conditions of the fast variable $\varphi _1$ for almost all initial conditions of the slow variables $(A_1, B_1)$, except for the case when they are in the close vicinity of the stable separatrix of the saddle point.

**Dependence of unsynchronized steady state on the sharpness parameter**$\mu$. The reduced system of Eq. (15) is convenient for analyzing the dependence of an unsynchronized steady state on the sharpness parameter $\mu$ of the boundary function. Especially in the limit of the hard boundary, $\mu \rightarrow 0$, the results can be obtained in analytical form. For the power boundary function (4), the stationary solutions of Eq. (15) at any $\mu$ can be demonstrated graphically. By equating the right hand sides of Eq. (15) to zero, we obtain two equations, $A_1=f_1(K)$ and $B_1=f_2(K)$, where

17 $$\begin{aligned} f_1(K) = \alpha \left[ 1+ \left( \frac{\int _{0}^{\pi }G_{-}(-K,-\varphi _1)\,{\text{d}}\varphi _1 }{\int _{-\pi }^{0}G_{+}(K,\varphi _1)\,{\text{d}}\varphi _1} \right) ^\frac{1}{\mu } \right] ^{-1}, \quad f_2(K) = \alpha \left[ 1+ \left( \frac{\int _{-\pi }^{0}G_{-}(K,\varphi _1)\,{\text{d}}\varphi _1}{\int _{0}^{\pi }G_{+}(-K,-\varphi _1)\,{\text{d}}\varphi _1} \right) ^\frac{1}{\mu }\right] ^{-1}. \end{aligned}$$

**Figure 2:**
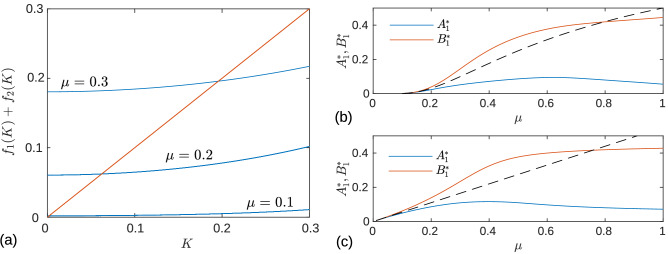
Sharpness parameter $\mu$ effect on synaptic weigths. (**a**) Graphical solution of the equation $K=f_1(K)+f_2(K)$ at different values of the parameter $\mu$ of the power boundary function (4). The stationary value of the variable *K* is defined by the intersection of the function $f_1(K)+f_2(K)$ with the identity line. The values of the parameters are the same as in Fig. 1. (**b**,**c**) Stationary solutions of Eq. (15) corresponding to unsynchronized state of the system in dependence of the sharpness parameter $\mu$ for (**b**) power (4) and (**c**) sigmoid (5) boundary functions. The dashed curves in (**b**,**c**) show the analytical predictions (22) and (23), respectively, which are valid for small values of the parameter $\mu$.

The stationary value of the variable $K=A_1+B_1$ satisfies the equation $K=f_1(K)+f_2(K)$. Figure 2a shows the graphical solution of this equation for different values of the parameter $\mu$. We see that the stationary point $K^*$ obtained as intersection of the curve $f_1(K)+f_2(K)$ with the identity line moves towards zero as $\mu$ is decreased. Since the synaptic weights $A_1$ and $B_1$ are always positive, the stationary values of $A_1^*$ and $B_1^*$ also move towards zero with decreasing $\mu$. This is demonstrated in Fig. 2b, were the dependence of the stationary values $A_1^*$ and $B_1^*$ on the parameter $\mu$ is depicted. Thus in the limit of the hard boundary $\mu \rightarrow 0$ the fixed point responsible for the unsynchronized state of the oscillators approaches the origin of the phase space, $(A_1^*, B_1^*) \rightarrow (0,0)$. This means that in the limit $\mu \rightarrow 0$, the oscillators becomes completely disconnected. To verify whether this property is universal, in Fig. 2c we plot the dependence of the stationary values of $A_1^*$ and $B_1^*$ on the parameter $\mu$ for the case of the sigmoid boundary function (5). We see that here the stationary values of $A_1^*$ and $B_1^*$ also tend to zero for $\mu \rightarrow 0$.

Now we generalize the above results for any continuous boundary function *F*(*x*), which for $0 \le x\le \alpha$ has the following properties: (i) *F*(*x*) is a monotonically increasing function, i.e., $F'(x)>0$; (ii) in the interval $0\le x \le \mu \ll \alpha$, the function increases sharply from $F(0)=0$ to $F(\mu ) \lesssim 1$, and in the interval $\mu \le x \le \alpha$, it slowly increases to a value $F(\alpha ) \approx 1$. In the examples above, we saw that for small $\mu$ the fixed point corresponding to the unsynchronized state was placed in the region $K \ll \Delta$. Here we assume that this condition is valid for any boundary function with the above properties and will show that the obtained results confirm this assumption. The assumption $K \ll \Delta$ allows us to simplify the Eq. (15) and treat the problem analytically. Under this assumption, the Eqs. (14) and (16) can be approximated as $T(K) \approx 2\pi /\Delta$ and $G_{\pm }(K,\varphi _1)\approx \exp \left( \varphi _1/\tau _{\pm }\right) /\Delta$, respectively. Substituting these expressions into Eq. (15) and evaluating the corresponding integrals, we get

18a $$\begin{aligned} \dot{A}_1= & {} \bigl \{ F(\alpha -A_{1})\tau _{+}\left[ 1-\exp (-\pi /\tau _{+})\right] -F(A_{1})\tau _{-}\left[ 1-\exp (-\pi /\tau _{-})\right] \bigr \}/2\pi , \end{aligned}$$

18b $$\begin{aligned} \dot{B}_1= & {} \bigl \{ F(\alpha -B_{1})\tau _{+}\left[ 1-\exp (-\pi /\tau _{+})\right] -F(B_{1})\tau _{-}\left[ 1-\exp (-\pi /\tau _{-})\right] \bigr \}/2\pi . \end{aligned}$$

We see that the equations for $A_{1}$ and $B_{1}$ are identical and independent of each other. This means that their stationary solutions coincide $A^*_1=B^*_1$ and it is enough to analyze only one of the two equations, say, the Eq. (18a). The stationary solution of Eq. (18a) satisfies:

19 $$\begin{aligned} \frac{F(A^{*}_{1})}{F(\alpha -A^{*}_{1})} = q, \end{aligned}$$

where

20 $$\begin{aligned} q = \frac{\tau _{+}\left[ 1-\exp (-\pi /\tau _{+})\right] }{\tau _{-}\left[ 1-\exp (-\pi /\tau _{-})\right] }, \end{aligned}$$

is a parameter depending on PDDP learning windows $\tau _+$ and $\tau _-$. At any $\tau _+ < \tau _-$, this parameter is less than one. Specifically, for $\tau _+=0.15$ and $\tau _-=0.3$ used in this paper, the value of this parameter is $q =0.5$. For small $\mu$, the function *F*(*x*) is close to 1 for all *x* except for a small region $0\le x \le \mu \ll \alpha$, where it sharply increases from $F(0)=0$ to $F(\mu ) \lesssim 1$. From this property it follows that for $q<1$ the Eq. (19) can be fulfilled only at $A^{*}_1 \sim \mu \ll \alpha$. Taking $F(\alpha -A^{*}_{1}) \approx F(\alpha ) \approx 1$, we get $F(A_1^*)\approx q$ and obtain the stationary solution of Eq. (18) in the form

21 $$\begin{aligned} A_1^*=B_1^*=F^{(-1)}(q), \end{aligned}$$

where $F^{(-1)}$ denotes the inverse of the function *F*. This solution is stable since $F'(A_1^*)>0$ and $F'(\alpha -A_1^*)>0$. Due to the assumptions made for the function *F*(*x*), the value of $F^{(-1)}(q)$ tends to zero for $\mu \rightarrow 0$. Therefore, for any boundary function with the above properties, the fixed point $(A_1^*, B_1^*)$ of the unsynchronized state approaches the origin of the phase plane when $\mu \rightarrow 0$. Choosing a sufficiently small value of $\mu$, we can always satisfy the condition $K \ll \Delta$, which was used to derive the Eq. (18).

The general Eq. (21) can be used to estimate unsynchronized stationary states for specific boundary functions (4) and (5) presented above. In the case of the power boundary function $F(x)=x^\mu$, we get

22 $$\begin{aligned} A_1^*=B_1^*=q^{1/\mu }. \end{aligned}$$

According to this law, the fixed point very quickly approaches the coordinate origin as $\mu$ decreases. In the case of a sigmoid boundary function $F(x)=\tanh (x/\mu )$, the fixed point approaches the origin linearly with decreasing $\mu$:

23 $$\begin{aligned} A_1^*=B_1^*=\mu\, {{\mathrm{atanh}}}\,(q). \end{aligned}$$

The dependencies (22) and (23) are shown by dashed curves in Fig. 2b,c, respectively. For small $\mu$ they are in good agreement with the results obtained by numerical solution of Eq. (15).

**Figure 3:**
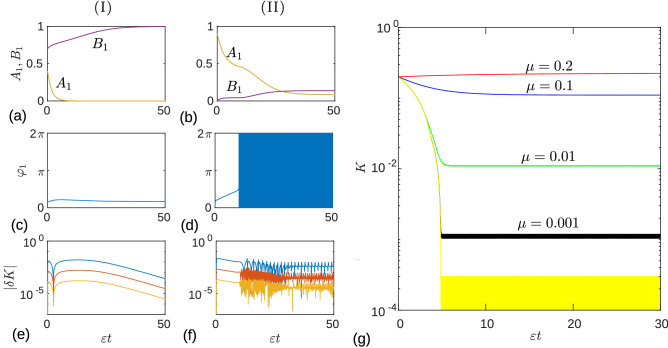
Dynamics of state variables of two oscillators under PDDP. Columns (I) and (II) represent two different initial conditions, which lead to different asymptotic modes: synchronized state (I) and unsynchronized state (II). (**a**,**b**) Dynamics of slow variables $A_1(t)$ and $B_1(t)$. (**c**,**d**) Dynamics of the phase difference $\varphi _1(t)$. The values of the parameters are the same as in Fig. 1 except for $\varepsilon =0.001$. (**e**,**f**) Dynamics of the absolute value $|\delta K (t)|$ of the deviation of the solution of the reduced system of Eqs. (11) and (15) from the solution of the original system of Eqs. (3) and (7) for different values of the parameter $\varepsilon$: 0.1 (blue), 0.01 (red) and 0.001 (orange). (**g**) Dynamics of the variable $K(t)=A_1(t)+B_1(t)$ obtained from Eqs. (3) and (7) for the sigmoid boundary function with different values of the parameter $\mu$: 0.2 (red), 0.1 (blue), 0.01 (green), and 0.001 (black). The curves move downward as the parameter $\mu$ decreases. The lower yellow curve corresponds to the Heaviside step boundary function. The initial conditions are the same as in the second column and $\varepsilon =0.001$.

Numerical examples in Fig. 3(I) and (II) columns show the dynamics of the system state variables for two different initial conditions that converge to different asymptotic modes: the synchronized state (I) and the unsynchronized state (II). The results are presented for the sigmoid boundary function (5) with the parameter $\mu =0.2$. Panels (a) and (b) show the dynamics of the slow variables $A_1(t)$ and $B_1(t)$ obtained from the original system of Eqs. (3) and (7), as well as the reduced system of Eqs. (11) and (15). For $\varepsilon =0.001$, these results are indistinguishable in the figure. In panel (a), the synaptic weight $A_1$ asymptotically vanishes ($A_1 \rightarrow 0$), and the synaptic weight $B_1$ reaches the maximum value ($B_1 \rightarrow \alpha =1$). The phases of the oscillators remain locked during this process, as can be seen from the dynamics of the phase difference $\varphi _1(t)$, shown in panel (c). In panel (b), both synaptic weights approach small values close to the value of the parameter $\mu =0.2$. Here the dynamics of the phase difference $\varphi _1(t)$ is more complex [see panel (d)]. The phases are locked when the sum $K=A_1+B_1$ of the synaptic weights is greater than the frequency detuning $\Delta =0.5$. When *K* becomes smaller than $\Delta$, the phase difference experiences fast oscillations. Panels (e) and (f) demonstrate the accuracy of the reduced Eqs. (11) and (15) depending on the parameter $\varepsilon$. The dynamics of the absolute value of the deviation $\delta K(t) = K_{\text {red}}(t)-K(t)$ in semi-log plot is shown for different values of the parameter $\varepsilon$: 0.1 (blue curves), 0.01 (red curves), and 0.001 (orange curves). Here *K*(*t*) is the sum of the synaptic weights $A_1(t)+B_1(t)$ obtained from the original system of Eqs. (3) and (7), and $K_{\text {red}}(t)$ is the same variable obtained from the reduced system of Eqs. (11) and (15). We see that the deviation $|\delta K|$ decreases in proportion to the $\varepsilon$ parameter.

Figure 3g shows the dynamics of the variable *K*(*t*) obtained from the original system of Eqs. (3) and (7), for fixed $\varepsilon =0.001$ and different values of the parameter $\mu$: 0.2 (red curve), 0.1 (blue curve), 0.01 (green curve), and 0.001 (black curve). These results are presented for the sigmoid boundary function $F(x)=\tanh (x/\mu )$. The lower yellow curve shows the result for the Heaviside step boundary function, $F(x)=H(x)$. The initial conditions are the same as in the (II) column, which correspond to the unsynchronized asymptotic state of the system. As expected from the above analytical results [see Eq. (23)], the asymptotic values of the variable *K* decrease in proportion to the $\mu$ parameter. Note that Eq. (23) was derived in the limit $\varepsilon \rightarrow 0$ and predicts stationary asymptotic values of *K*. Here we observe small amplitude oscillations in the asymptotic dynamics due to the finite value of $\varepsilon$. The oscillations with a small amplitude of order $\varepsilon$ remain also in the case of the Heaviside step boundary function (see the lower yellow curve in the figure).

To conclude this section, we formulate the main results concerning the asymptotics of two coupled oscillators with PDDP. Below, we will be interested in the case of small parameters $\varepsilon$ and $\mu$, since this case allows to predict the number of possible states in a star network with an arbitrary number of leaves. In this case the system of two oscillators can demonstrate bistability with coexisting synchronized and unsynchronized attractors. The synchronized attractor is characterized by a unidirectional coupling, in which directional link from a slower oscillator to a faster oscillator is disconnected, $A_1=0$, and the synapse associated with the directional link from a faster oscillator to a slower oscillator is maximal, $B_1=\alpha$. This attractor exists only when the maximum value $\alpha$ of the synaptic weight is greater than the frequency mismatch $\Delta$ between the oscillators. The unsynchronized attractor is characterized by low values of both synaptic weights $A_1$ and $B_1$. In the limit $\mu \rightarrow 0$ and $\varepsilon \rightarrow 0$, the oscillators in the unsynchronized state are completely disconnected, $A_1=B_1=0$. In the next section, we will use these results to predict the asymptotic behavior of multi-leaf star networks.

## Results

We show that a star network with *N* leaves described by Kuramoto-type phase oscillators Eq. (1), with synaptic weights satisfying the PDDP rule (3), can evolve into $2^N$ various stable configurations. This result was obtained under the assumption of small parameters $\varepsilon$ and $\mu$. We use the analytic results obtained for two oscillators to predict the asymptotic behavior of an arbitrary star network. A generalization for an *N*-leaf star network is obtained by gradually enlarging the number of leaves. We prove our prediction numerically for the cases of two, three, and nine leaves.

For simplicity, we assume that there are no oscillators with coinciding natural frequencies. Then, without loss of generality, let us number the natural frequencies of the leaves in ascending order:

24 $$\begin{aligned} \omega _1<\omega _2< \cdots < \omega _N. \end{aligned}$$

We will consider all possible options for choosing the natural frequency $\omega _0$ of the hub, which can fall into different frequency intervals of the leaves, i.e., it can satisfy $N+1$ various inequalities: $\omega _0<\omega _1$, $\omega _j<\omega _0<\omega _{j+1}$ for $j=1,\ldots ,N-1$, and $\omega _N<\omega _0$. Finally, we assume that the limiting value $\alpha$ of synaptic weights is greater than the frequency mismatches $\Delta _{j}=|\omega _0-\omega _j|$ between the hub and all leaves:

25 $$\begin{aligned} \alpha >\Delta _{j}, \quad j=1,\ldots , N. \end{aligned}$$

The last inequalities are a generalization of the necessary condition (13) for the existence of synchronized states.

**Figure 4:**
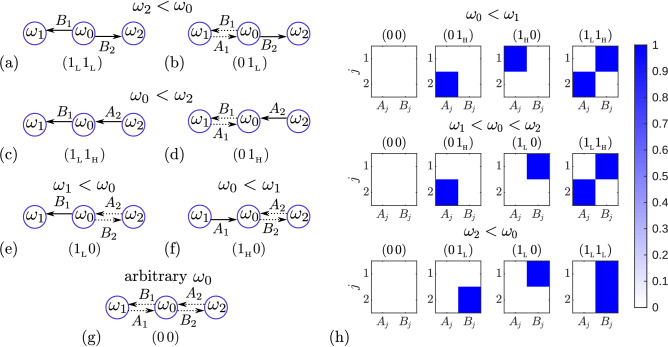
Asymptotic configurations for a two-leaf star network. (**a**–**g**) Predicted configurations. Solid arrows show the unidirectional coupling with maximum synaptic weight that results in synchronization between the leaf and the hub. Dashed arrows correspond to a weak synaptic connections, so the leaf is not synchronized with the hub. The required frequency inequality is indicated above each configuration. The two characters in brackets under each configuration represent its code. (**h**) Distributions of color-coded asymptotic values of coupling weights $A_j$ and $B_j$ for a two-leaf star network, obtained by integrating Eqs. (3) and (6). The results presented in three rows correspond to three different possibilities of the hub frequency to fall into different frequency intervals of the leaves: $\omega _0<\omega _1$ (top row), $\omega _1<\omega _0<\omega _2$ (middle row), and $\omega _2<\omega _0$ (bottom row). The top row of (**h**) (from left to right) match configurations (**g**,**b**,**e**,**a**). For the middle and bottom rows, the corresponding configurations are, respectively, as follows: (**g**,**d**,**e**,**c**) and (**g**,**d**,**f**,**c**). All frequencies are taken from the array (0.55, 0.85, 1). The parameter $\varepsilon =0.001$, and the parameter $\mu = 0.01$ corresponds to the sigmoid boundary function (5). The values of the parameters $\alpha$, $\tau _+$ and $\tau _-$ are the same as in Fig. 1.

**Two-leaf star network.** We begin our analysis with a two-leaf star network. We assume that each leaf in the network can be either synchronized or unsynchronized with the hub. We also assume that the fastest oscillator always wins in the synchronization process, so that unidirectional links from the fastest oscillator to the slower oscillators are established with the maximum synaptic weights. Since the fastest oscillator dominates the slower oscillators, no more than one synchronized link can go to the hub. Using these assumptions, we constructed seven possible configurations, shown in Fig. 4a–g. Solid arrows correspond to the synchronized states of the oscillators, in which the connection is unidirectional with the maximum synaptic weight. Dotted arrows correspond to unsynchronized states between oscillators, in which synaptic weights in both directions are small. We assume that they vanish in the limit $\varepsilon \rightarrow 0$ and $\mu \rightarrow 0$. The presented configurations can be realized only when certain frequency inequalities are met. These inequalities are written above each configuration. The inequalities were obtained using the Eqs. (1) and (3). Below we illustrate this with examples of three configurations (a), (c) and (e). In these examples, we also show that these configurations satisfy the Eqs. (1) and (3).

In configuration (a), the coupling weights $A_1$ and $A_2$ are zeros, and both leaves are synchronized with the hub. In this case, the phases of the oscillators satisfy the equations:

26a $$\begin{aligned} \dot{\theta }_{0}= & {} \omega _{0}, \end{aligned}$$

26b $$\begin{aligned} \dot{\theta }_{1}= & {} \omega _{1}+B_{1}\sin \left( {\theta }_{0} -\theta _{1}\right) ,\end{aligned}$$

26c $$\begin{aligned} \dot{\theta }_{2}= & {} \omega _{2}+B_{2}\sin \left( \theta _{0}-\theta _{2}\right) . \end{aligned}$$

The hub is not influenced by leaves, so the instantaneous frequencies of the leaves must be equal to the natural frequency of the hub, $\dot{\theta }_{1}=\dot{\theta }_{2}=\omega _0$, to ensure synchronization. This leads to two equations for the phase differences as defined by Eq. (2) [$\varphi _{1,2}={\theta }_{0}-{\theta }_{1,2}$]: $\sin \left( \varphi _{1,2}\right) =\left( \omega _{0}-\omega _{1,2}\right) /B_{1,2}$. From Eq. (3a) we conclude that the phase differences $\varphi _{1,2}$ must be in the interval $(-\pi ,0)$ in order to satisfy the conditions $A_1=A_2=0$. Then from the stationary solution of the Eq. (3b), we get $B_1=B_2=\alpha$, and from the above equations for the phase differences, we obtain two inequalities: $\omega _{1} < \omega _0$ and $\omega _{2} < \omega _0$. In Fig. 4a–g we have presented only the second inequality corresponding to this configuration, since the first inequality holds due to the assumption (24).

As a second illustrative example, consider configuration (c). Here, as in the previous example, all oscillators are synchronized, but now $A_1$ and $B_2$ are zeros, and the Eq. (1) for the phases can be written as:

27a $$\begin{aligned} \dot{\theta }_{0}= & {} \omega _{0}-A_{2}\sin \left( {\theta }_{0}-\theta _{2}\right) ,\end{aligned}$$

27b $$\begin{aligned} \dot{\theta }_{1}= & {} \omega _{1}+B_{1}\sin \left( {\theta }_{0}-\theta _{1}\right) ,\end{aligned}$$

27c $$\begin{aligned} \dot{\theta }_{2}= & {} \omega _{2}. \end{aligned}$$

The second leaf is not influenced by the hub and it oscillates freely with its natural frequency $\omega _2$. To ensure synchronization between all oscillators, we must require $\dot{\theta }_{0}=\dot{\theta }_{1}=\omega _2$. As a result, we get two equations for the phase differences $\varphi _{1,2}$: $\sin \left( \varphi _{1}\right) =\left( \omega _{2}-\omega _{1}\right) /B_{1}$ and $\sin \left( \varphi _{2}\right) =\left( \omega _{0}-\omega _{2}\right) /A_{2}$. It follows from the first equation that the phase difference $\varphi _1$ is in the interval $(0,\pi )$, since $\omega _2>\omega _1$. This provides $A_1=0$ and $B_1=\alpha$ as stationary solutions to the Eqs. (3a) and (3b). To satisfy the condition $B_2=0$, we must require that the phase difference $\varphi _{2}$ is in the interval $(-\pi ,0)$. Then from the stationary solution of the Eq. (3b) we get $A_2=\alpha$, and from the above equations for the phase difference $\varphi _{2}$ we obtain the inequality $\omega _0<\omega _2$, which gives the necessary condition for this configuration.

Our third example is configuration (e). Here, the first leaf is synchronized with the hub and the second leaf is not synchronized. Considering that $A_1=0$ and the weights $A_2$ and $B_2$ are small, the Eq. (1) for the phases take the form:

28a $$\begin{aligned} \dot{\theta }_{0}= & {} \omega _{0}+O(A_2),\end{aligned}$$

28b $$\begin{aligned} \dot{\theta }_{1}= & {} \omega _{1}+B_{1}\sin \left( \theta _{0}-\theta _{1}\right) ,\end{aligned}$$

28c $$\begin{aligned} \dot{\theta }_{2}= & {} \omega _{2}+O(B_2). \end{aligned}$$

Here $O(A_2)$ and $O(B_2)$ are small terms proportional to the weights $A_2$ and $B_2$ respectively, which we assume to be zero for $\varepsilon \rightarrow 0$ and $\mu \rightarrow 0$. If we neglect these terms, then we will see that the hub and the second leaf freely oscillate with their natural frequencies. The synchronization condition of the first leaf with the hub, $\dot{\theta }_{1}=\omega _0$, leads to the equation $\sin \left( \varphi _{1}\right) =\left( \omega _{0}-\omega _1\right) /B_{1}$. From the Eq. (3a) it follows that the phase difference $\varphi _{1}$ must be in the interval $(-\pi ,0)$ in order to satisfy the condition $A_1=0$. Then from the stationary solution of the Eq. (3b), we get $B_1=\alpha$, and from the above equation for the phase difference $\varphi _1$, we obtain the inequality $\omega _{1} < \omega _0$, which is the necessary condition for the realization of configuration (e).

For further classification of asymptotic states, it is convenient to introduce the following notations. We encode each asymptotic configuration of an N-leaf network as an array of *N* characters $(s_1 s_2 \ldots s_N)$, where $s_j$ defines the state of the *j*th leaf. Each element $s_j$ of the array is represented by one of three possible symbols: “0” (zero), “$1_{\mathrm {H}}$” (one with subscript H), and “$1_{\mathrm{L}}$” (one with subscript L). The symbol “0” denotes an unsynchronized state of the leaf with small (vanishing for $\varepsilon \rightarrow 0$ and $\mu \rightarrow 0$) synaptic weights. The symbols “$1_{\mathrm {H}}$” and “$1_{\mathrm {L}}$” represent the synchronized state with maximum coupling strength directed to the hub and leaf, respectively. The corresponding codes are written under each configuration in Fig. 4.

All asymptotic configurations shown in Fig. 4a–g were numerically validated for different sets of hub and leaves frequencies. Using these configurations, we can predict all possible asymptotic states of a two-leaf network for any given value of the hub frequency when it falls into different frequency intervals of the leaves. When the hub frequency is less than the frequencies of both leaves, $\omega _0 <\omega _1$, then, depending on the initial conditions, the configurations (c), (d), (f) and (g) can be asymptotically attained. When the hub frequency falls within the interval between leaf frequencies, $\omega _1<\omega _0<\omega _2$, the admissible asymptotic configurations are (c), (d), (e), and (g). Finally, when the hub frequency is greater than the frequencies of both leaves, $\omega _2<\omega _0$, we identify (a), (b), (e), and (g) as admissible configurations. Thus, at any value of the hub frequency, a two-leaf star network can asymptotically reach four different configurations, characterized by different values of synaptic weights.

In Fig. 4h, we confirm the above statement with a specific numerical example. Taking different initial conditions, we integrated the system of Eqs. (3) and (6) over a long period of time until the values of the weights $A_j$ and $B_j$ became saturated, and then displayed these values in color code. The results presented in three rows correspond to three different possibilities of the hub frequency to fall into different frequency intervals of the leaves: $\omega _0<\omega _1$ (top row), $\omega _1<\omega _0<\omega _2$ (middle row), and $\omega _2<\omega _0$ (bottom row). In all cases, four different asymptotic states were found, as predicted above. On each row, we order the asymptotic states so that we get similar sequences of configuration codes. If we remove the subscripts at “$1_{\mathrm {H}}$” and “$1_{\mathrm {L}}$” in these codes, we get an identical sequences of configuration codes for each row: $(0\,0)$, $(0\,1)$, $(1\,0)$, and $(1\,1)$. This is a binary sequence of natural numbers from 0 to 3, which gives us a convenient way to index the various asymptotic configurations for any given hub frequency. We will use this way of numbering different asymptotic configurations when considering star networks with a larger number of leaves.

**Figure 5:**
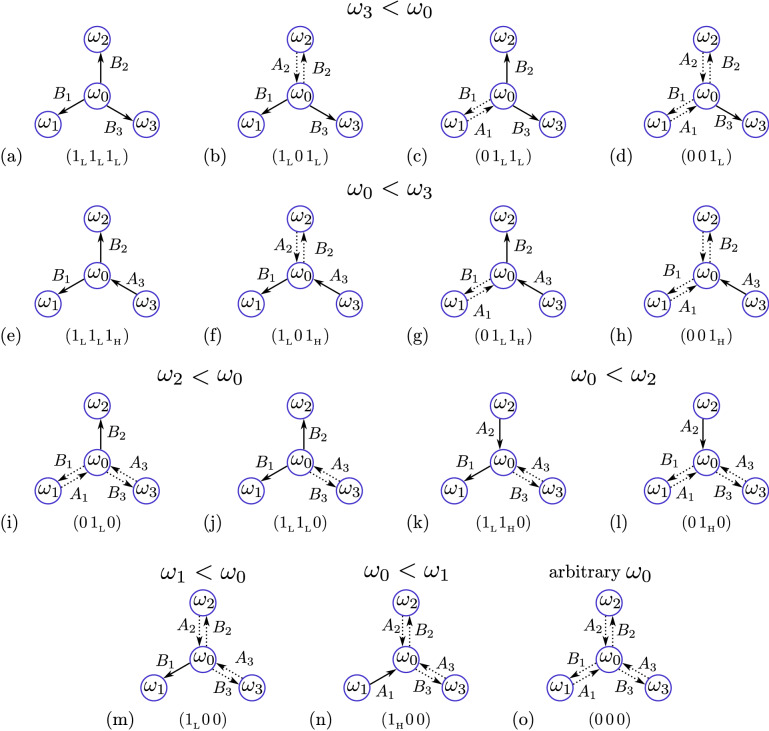
(**a-o**) Possible asymptotic configurations for a three-leaf star network. See capture to Fig. 4 for details.

**Three-leaf star network.** The classification of various asymptotic states in a three-leaf star network can be done in the same way as for a two-leaf star network. Using arguments similar to those given above, we have constructed fifteen possible asymptotic configurations of the three-leaf star network, which are shown in Fig. 5. They are designated by letters from (a) to (o). The three characters in brackets below each configuration represent its code. The required frequency inequality is indicated above each configuration.

Figure 5 allows predicting all possible asymptotic states of a three-leaf star network for any given value of the hub frequency when it falls into different frequency intervals of the leaves. In all cases, there are eight different admissible asymptotic states presented in Table 1. The first column lists four different possible inequalities for the hub frequency, and the next eight columns show the codes of the corresponding allowed configurations. For example, when the hub frequency falls within the interval between the frequencies of the second and third leaves, $\omega _2<\omega _0<\omega _3$, the codes listed from left to right in the third row correspond to configurations (o), (h), (i), (g), (m), (f), (j), and (e) in Fig. 5. We order the configurations in the same way as in the case of a two-leaf star network. After removing the subscripts at “$1_{\mathrm {H}}$” and “$1_{\mathrm {L}}$” in the configuration codes, we get identical binary sequence of natural numbers from 0 to 7 in all rows of the Table 1. We use this numbers to index the corresponding asymptotic configurations for any given hub frequency.

**Table 1:** Theoretically predicted asymptotic configurations of a three-leaf star network for four different possibilities of the hub frequency to fall into different frequency intervals of the leaves.

$\omega _{0}<\omega _{1}$	$(0\,0\,0)$	$(0\,0\,1_{{\mathrm {H}}})$	$(0\,1_{{\mathrm {H}}}0)$	$(0\,1_{{\mathrm {L}}}1_{{\mathrm {H}}})$	$(1_{{\mathrm {H}}}0\,0)$	$(1_{{\mathrm {L}}}0\,1_{{\mathrm {H}}})$	$(1_{{\mathrm {L}}}1_{{\mathrm {H}}}0)$	$(1_{{\mathrm {L}}}1_{{\mathrm {L}}}1_{{\mathrm {H}}})$
$\omega _{1}<\omega _{0}<\omega _{2}$	$(0\,0\,0)$	$(0\,0\,1_{{\mathrm {H}}})$	$(0\,1_{{\mathrm {H}}}0)$	$(0\,1_{{\mathrm {L}}}1_{{\mathrm {H}}})$	$(1_{{\mathrm {L}}}0\,0)$	$(1_{{\mathrm {L}}}0\,1_{{\mathrm {H}}})$	$(1_{{\mathrm {L}}}1_{{\mathrm {H}}}0)$	$(1_{{\mathrm {L}}}1_{{\mathrm {L}}}1_{{\mathrm {H}}})$
$\omega _{2}<\omega _{0}<\omega _{3}$	$(0\,0\,0)$	$(0\,0\,1_{{\mathrm {H}}})$	$(0\,1_{{\mathrm {L}}}0)$	$(0\,1_{{\mathrm {L}}}1_{{\mathrm {H}}})$	$(1_{{\mathrm {L}}}0\,0)$	$(1_{{\mathrm {L}}}0\,1_{{\mathrm {H}}})$	$(1_{{\mathrm {L}}}1_{{\mathrm {L}}}0)$	$(1_{{\mathrm {L}}}1_{{\mathrm {L}}}1_{{\mathrm {H}}})$
$\omega _{3}<\omega _{0}$	$(0\,0\,0)$	$(0\,0\,1_{{\mathrm {L}}})$	$(0\,1_{{\mathrm {L}}}0)$	$(0\,1_{{\mathrm {L}}}1_{{\mathrm {L}}})$	$(1_{{\mathrm {L}}}0\,0)$	$(1_{{\mathrm {L}}}0\,1_{{\mathrm {L}}})$	$(1_{{\mathrm {L}}}1_{{\mathrm {L}}}0)$	$(1_{{\mathrm {L}}}1_{{\mathrm {L}}}1_{{\mathrm {L}}})$

The first column lists the possible inequalities for the hub frequency, and the next eight columns show the codes of the corresponding allowed configurations.

**Figure 6:**
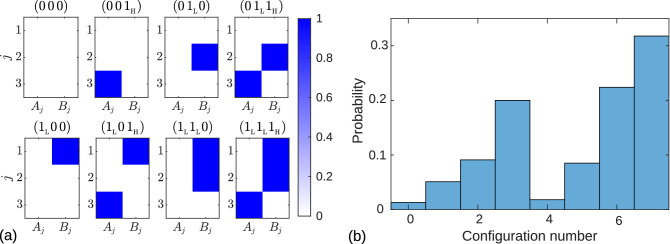
Results of three leaf star modeling. (**a**) Distributions of color-coded asymptotic values of coupling weights $A_j$ and $B_j$ for a three-leaf star network, obtained by integrating Eqs. (3) and (6) with different initial conditions. The leaves natural frequencies are $(\omega _1, \omega _2, \omega _3)$ = (0.55, 0.7, 1). The hub natural frequency $\omega _0=0.85$ satisfies the inequality $\omega _2<\omega _0<\omega _3$. The resulting distributions are labeled with codes that agree with the theoretically predicted asymptotic configurations shown in the third column of Table 1. (**b**) Probability distribution of eight different asymptotic configurations for a three-leaf star network, when the hub frequency is in the interval between the frequencies of the second and third leaves, $\omega _2<\omega _0<\omega _3$. In panel (**a**), the configurations are numbered according to the decimal representation of the binary codes shown above each pattern in panel (**a**) (see main text for details). The distribution is constructed using 1000 randomly selected initial conditions for Eqs. (3) and (6). The initial values of the weights $A_j$ and $B_j$ were randomly and independently taken from the uniform distribution $[0, \alpha ]$.

All asymptotic configurations presented in Table 1 were numerically validated for different sets of hub and leaves frequencies. An example of numerical testing of asymptotic states of a tree-leaf star network, when the hub frequency is in the interval between the frequencies of the second and third leaves, $\omega _2<\omega _0<\omega _3$, is shown in Fig. 6a. By integrating the system of Eqs. (3) and (6) with many different initial conditions over a long period of time, we obtained eight different asymptotic configurations, as predicted in the third column of the Table 1. The probability distribution of the various asymptotic configurations, numbered as described above, is shown in Fig. 6b. This graph was constructed using 1000 randomly selected initial conditions. The initial values of the slow variables $A_j$ and $B_j$ were randomly and independently taken from uniform distribution $[0, \alpha ]$. Each of the 1000 numerical experiments resulted in one of eight predicted asymptotic configurations, and no other asymptotic solutions were observed. For given parameter values, configuration $(1_{{\mathrm {L}}}1_{{\mathrm {L}}}1_{{\mathrm {H}}})$ with all synchronized oscillators (Fig. 5e) is most likely, and configuration $(0\,0\,0)$ with all unsynchronized oscillators (Fig. 5o) is the least likely.

*N***-leaf star network.** The results obtained for two- and three-leaf star networks can be generalized for a star network with an arbitrary number of leaves. Assuming that each leaf in a *N*-leaf star network can be either synchronized or unsynchronized with the hub, we can construct $2^N$ different asymptotic configurations for any given hub frequency. Let us denote the code of the *n*th asymptotic configuration of an *N*-leaf star network as ${\mathcal {C}}_n^{(k)}$, where *n* is a natural number that varies from 0 to $2^N-1$, and the superscript (*k*) identifies a specific frequency range of the hub. We assign $k=1$ when the hub frequency is less than the lowest frequency of the leaves $\omega _0<\omega _1$. The values $k=2,\ldots , N$ correspond to the inequalities $\omega _{k-1}<\omega _0<\omega _k$. Finally, we assign $k=N+1$, when the hub frequency is greater than the highest frequency of the leaves, $\omega _N<\omega _0$. Summarizing the results of sections dedicated to two and three leaf networks, we found that the code ${\mathcal {C}}_n^{(k)}$ of the *n*th asymptotic configuration for any *N* can be written as follows. First, we represent the decimal number *n* in binary: $n=(d_{N-1} d_{N-2}\ldots d_0)_2=\sum _{j=0}^{N-1}d_j2^j$, were $d_j$ are digits equal to 0 or 1. Then the rightmost digit 1 in the positions $p \ge k$ of the array $(d_{N-1} d_{N-2}\ldots d_0)$ is assigned the subscript H. After that, all the remaining digits 1 in the array are assigned the subscript L, and this gives us the required code ${\mathcal {C}}_n^{(k)}$. To demonstrate these rules, let’s write the code for the third asymptotic configuration of a three-leaf star network when the hub frequency is in the range $\omega _1<\omega _0<\omega _2$. In this case $k=2$, $N=3$ and $n=3$. The number *n* in binary is $(0\, 1\, 1)$. Assigning the rightmost digit 1 in the positions $p\ge 2$ to the subscript H, and the remaining digits 1 to the subscript L, we obtain the code ${\mathcal {C}}_3^{(2)}=(0\,1_{{\mathrm {L}}} 1_{{\mathrm {H}}})$, which is the same as shown in the second row of the fifth column of Table 1. As an example of applying the above rules to a more complex case, we present the code of the 25th asymptotic configuration of a five-leaf star network when the hub frequency is in the range $\omega _4<\omega _0<\omega _5$:

29 $$\begin{aligned} {\mathcal {C}}_{25}^{(5)}=(1_{{\mathrm {L}}} 1_{{\mathrm {L}}} 0 \, 0 \, 1_{{\mathrm {H}}}). \end{aligned}$$

To verify the validity of the predicted asymptotic states in a star network with a large number of leaves, we will consider the dynamics of a 2*N*-dimensional state vector

30 $$\begin{aligned} {\mathbf {R}}(t)=(A_{1},A_{2},\ldots ,A_{N},B_{1},B_{2},\ldots ,B_{N})^T \end{aligned}$$

in the complete phase space of slow variables. By integrating the system of Eqs. (3) and (6) with different initial conditions, we will check whether the state of the network approaches the predicted asymptotic states. The state vector

31 $$\begin{aligned} {\mathbf {R}}_n^{(k)}=(A_{1}^*,A_{2}^*,\ldots ,A_{N}^*,B_{1}^*,B_{2}^*,\ldots ,B_{N}^*)^T \end{aligned}$$

of the *n*th predicted asymptotic configuration, determined by its code ${\mathcal {C}}_{n}^{(k)}=(s_1 s_2 \ldots s_N)$ is constructed as follows. In the first half of the vector components, we assign $A_j^*=\alpha$ if the corresponding character $s_j$ in the code is “$1_{{\mathrm {H}}}$”, and we assign $A_j^*=0$ if the character $s_j$ is “$1_{{\mathrm {L}}}$” or “0”. In the second half of the vector components, we assign $B_j^*=\alpha$ if the character $s_j$ is “$1_{{\mathrm {L}}}$”, and we assign $B_j^*=0$ if the character $s_j$ is “$1_{{\mathrm {H}}}$” or “0”. For example, the state vector of the 25th predicted asymptotic configuration in a five-leaf star network defined by the code Eq. (29) is:

32 $$\begin{aligned} {\mathbf {R}}_{25}^{(5)}=(0,0,0,0,\alpha ,\alpha ,\alpha ,0,0,0)^T. \end{aligned}$$

**Figure 7:**
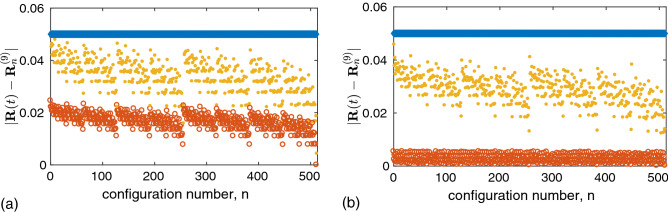
Numerical simulation of Eqs. (3) and (6) for a nine-leaf star network with 512 different initial conditions ${\mathbf {R}}(0)$, each of which is close to the state ${\mathbf {R}}_n^{(9)}$ of a particular predicted asymptotic configuration with number $n=0,\ldots ,511$. Panels (**a**) and (**b**) correspond to the sigmoid boundary function with $\mu =0.01$ and the Heaviside step boundary function, respectively. The frequencies $(\omega _1, \ldots , \omega _8, \omega _0, \omega _9)$, written in ascending order, are equidistantly distributed in the interval [0.6, 1]. The states ${\mathbf {R}}(0)$ are chosen so that the initial distances $|{\mathbf {R}}(0)-{\mathbf {R}}_n^{(9)}|$ shown in blue squares are the same for all configurations. The yellow dots show the values of the corresponding distances $|{\mathbf {R}}(t)-{\mathbf {R}}_n^{(9)}|$ at time $t=300$, and the red circles at time $t=76{,}000$. Parameter values: $\varepsilon = 0.001$, $\tau _+ = 0.15$, $\tau _-=0.3$, and $\alpha = 1$.

In Fig. 7, we confirm the validity of the predicted asymptotic states for a nine-leaf star network when the hub frequency is in the range $$\omega _8<\omega _0<\omega _9$$. Panel (a) shows the results obtained with the sigmoid boundary function (5) at $$\mu =0.01$$, and panel (b) corresponds to the Heaviside step boundary function. To numerically check the existence of the predicted asymptotic configurations in the system of Eqs. (3) and (6), we prepared 512 different initial states $${\mathbf {R}}(0)$$ so that they are at the same small distance $$|{\mathbf {R}}(0)-{\mathbf {R}}_n^{(9)}|=0.05$$ from different theoretical configurations defined by state vectors $${\mathbf {R}}_n^{(9)}$$, $$n=0,\ldots 
,511$$. These initial distances are depicted as blue squares on the top horizontal line. The evolution of the distances obtained by integrating the Eqs. (3) and (6) for the initial conditions chosen in this way is presented by two snapshots. The yellow dots show the values of the corresponding distances $$|{\mathbf {R}}(t)-{\mathbf {R}}_n^{(9)}|$$ at time $$t=300$$, and the red circles at time $$t=76{,}000$$. We see that for both boundary functions, the distances from the predicted states decrease with time, which means that these states are stable asymptotic network configurations. However, the distances (except for the case $$n=511$$) do not vanish even after a fairly long time. This is because we integrate the Eqs. (3) and (6) for finite values of the parameters $$\mu$$ and $$\varepsilon$$, while our theoretical prediction of asymptotic states is based on assumption $$\mu \rightarrow 0$$ and $$\varepsilon \rightarrow 0$$. Exceptional configuration $$n=511$$ corresponds to the state with all synchronized oscillators. The distance from this state tends to zero, since this state is an exact solution of the Eqs. (3) and (6) even for finite $$\mu$$ and $$\varepsilon$$. All other predicted asymptotic states are approximate solutions of the system of Eqs. (3) and (6). Note that the Heaviside boundary function results in better match of the asymptotic solutions of the Eqs. (3) and (6) with the predicted ones than the sigmoid function; the final distances $$|{\mathbf {R}}(t)-{\mathbf {R}}_n^{(9)}|$$ at time $$t=76{,}000$$ in Fig. 7b are smaller than in Fig. 7a. This is due to the fact that in the case of the sigmoid boundary function, both parameters $$\mu$$ and $$\varepsilon$$ are finite, and in the case of the Heaviside boundary function only $$\varepsilon$$ is finite.

## Discussion

We analyzed the multistability in a star network of Kuramoto-type phase oscillators in the presence of phase-difference-depended plasticity. A star-type network is the simplest network model that captures many of the typical properties of real-world networks; it can be considered an essential building block of neural networks^29–31^. The relative simplicity of the network allowed us to accurately estimate the number of possible asymptotic configurations which can be attained during the plastic evolution of the network. We have shown that, depending on the initial conditions, an *N*-leaf star plastic network can evolve into $2^N$ various stable configurations characterized by different values of the coupling strength between the hub and the leaves. This result is independent of the relative magnitude of the natural frequency of the hub in relation to the natural frequencies of the leaves. We classified the various asymptotic states of the network by introducing configuration codes. The code is an array of *N* symbols that define the asymptotic state of each leaf. Each leaf can be synchronized or unsynchronized with the hub.

Results for an arbitrary *N*-leaf star network were obtained by generalizing the results derived from the analysis of networks with a small number of leaves. We started our analysis from a simple model consisting of two oscillators. Using the small parameter $\varepsilon$, which determines the ratio of the characteristic time scales of phase variables and synaptic weights, we obtained a reduced system of two differential equations for slowly varying synaptic weights. These equations were treated analytically. We have shown that this system is bistable with coexisting synchronized and unsynchronized attractors. The synchronized attractor is characterized by a unidirectional coupling, in which the directional link from a slower oscillator to a faster oscillator is disconnected, and the synapse associated with the directional link from a faster oscillator to a slower oscillator is maximal. The unsynchronized attractor is characterized by low values of both synaptic weights. In the limit of the hard boundary, when the sharpness parameter $\mu$ of the PDDP boundary function tends to 0, the oscillators in the unsynchronized state are completely disconnected. Using these results, we predicted all possible asymptotic states for a two- and three-leaf star networks and confirmed our prediction with numerical examples. Next, we generalized our prediction for an arbitrary *N*-leaf star network. In the limit of two small parameters $\varepsilon \rightarrow 0$ and $\mu \rightarrow 0$, we developed an algorithm for constructing $2^N$ different asymptotic configurations. As an example, we demonstrated the applicability of this algorithm to a nine-leaf star network. Using our algorithm, we predicted 512 different asymptotic configurations and numerically proved their existence in a network model with small values of the parameters $\varepsilon$ and $\mu$.

Multistability of neuronal networks is relevant to a number of applications. For instance, as shown numerically in Refs.^9,40–42^, plasticity-mediated multistability enables desynchronizing and/or decoupling stimulus patterns to move neuronal networks from dynamic states characterized by abnormally strong synchrony and correspondingly increased synaptic weights to dynamic states with reduced synchrony and reduced synaptic strengths. Coordinated Reset (CR) stimulation^43^ is a spatio-temporally patterned desynchronization stimulation protocol which was computationally designed to induce cumulative and long-lasting therapeutic desynchronizing effects by shifting neuronal networks from abnormally synchronized attractors with strong synaptic weights to desynchronized attractors with reduced synaptic weights^9,44^. Cumulative and long-lasting therapeutic and desynchronizing effects were observed in Parkinson’s disease (PD) patients^45^ and Parkinsonian monkeys^46,47^ treated with CR-deep brain stimulation as well as in PD patients treated with vibrotactile CR stimulation delivered to the fingertips^48,49^. By the same token, acoustic CR stimulation induced cumulative and long-lasting reduction of tinnitus loudness and tinnitus annoyance along with a reduction of tinnitus-related abnormal cortical synchrony^50,51^.

Our analysis strategy, extending analytical results obtained in small systems to larger networks, enables thorough analysis of plastic mechanisms of high-dimensional dynamical systems, as e.g. also performed by applying two-neuron loop analysis in the context of recurrent networks of oscillatory neurons with propagation delays^11^. As a further plan of this study, it is interesting to check the universality of the results obtained. The question is whether the number of different asymptotic states in a star network remains the same if we replace phase oscillators with realistic neuron models and use the more precise STDP rule. A computationally attractive model for this problem would be a star network of integrate-and-fire neurons, which enables event-driven simulations^20^. It is also interesting to extend this study to more complex network topologies. The existence of desynchronized states with low coupling weights allows us to assume that our model will lead to the appearance of multi-cluster states in complex network topologies, as was observed in Refs.^52,53^. The effect of noise is also of great interest as it may induce new states in the network^27,54^.
