# Publisher Correction: Multistability in a star network of Kuramoto-type oscillators with synaptic plasticity

**DOI:** 10.1038/s41598-021-98189-0

**Published:** 2021-09-14

**Authors:** Irmantas Ratas, Kestutis Pyragas, Peter A. Tass

**Affiliations:** 1 Center for Physical Sciences and Technology, 10257 Vilnius, Lithuania; 2 Department of Neurosurgery, School of Medicine, Stanford University, Stanford, CA 94305 USA

Correction to: *Scientific Reports* 10.1038/s41598-021-89198-0, published online 10 May 2021

The original version of this Article contained errors.

In Figure 7, the x-axis labels, “configuration number, n” did not display correctly and was incorrectly given as “configuration num er, n”.

The original Figure 7 and accompanying legend appear below.

**Figure 7:**
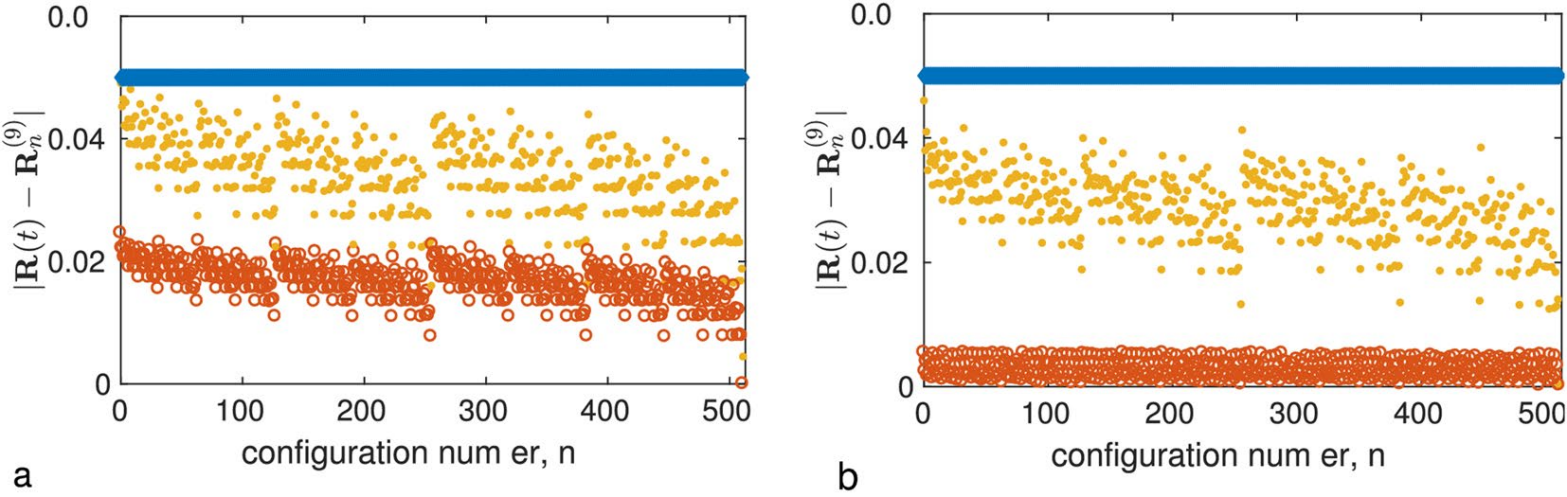
Numerical simulation of Eqs. (3) and (6) for a nine-leaf star network with 512 different initial conditions ${\mathbf {R}}(0)$, each of which is close to the state ${\mathbf {R}}_n^{(9)}$ of a particular predicted asymptotic configuration with number $n=0,\ldots ,511$. Panels (**a**) and (**b**) correspond to the sigmoid boundary function with $\mu =0.01$ and the Heaviside step boundary function, respectively. The frequencies $(\omega _1, \ldots , \omega _8, \omega _0, \omega _9)$, written in ascending order, are equidistantly distributed in the interval [0.6, 1]. The states ${\mathbf {R}}(0)$ are chosen so that the initial distances $|{\mathbf {R}}(0)-{\mathbf {R}}_n^{(9)}|$ shown in blue squares are the same for all configurations. The yellow dots show the values of the corresponding distances $|{\mathbf {R}}(t)-{\mathbf {R}}_n^{(9)}|$ at time $t=300$, and the red circles at time $t=76{,}000$. Parameter values: $\varepsilon = 0.001$, $\tau _+ = 0.15$, $\tau _-=0.3$, and $\alpha = 1$.

Additionally, there were errors in the Reference list.

Reference 42 was incorrectly given as:

42. Khaledi Nasab, A., Kromer, J. & Tass, P. Long-lasting desynchronization of plastic neural networks by random reset stimulation. *Front. Physiol.* **(in press)** (2020).

Reference 49 was incorrectly given as:

49. Pfeifer, K. J. *et al.* Coordinated reset vibrotactile stimulation induces sustained cumulative benefits in Parkinson’s disease. *Front. Physiol.* **(Under review)** (2021).

The correct References are listed below:

42. Khaledi Nasab, A., Kromer, J. & Tass, P. Long-lasting desynchronization of plastic neural networks by random reset stimulation. *Front. Physiol.* **11,** 622620. 10.3389/fphys.2020.622620 (2021).

49. Pfeifer, K. J. *et al.* Coordinated reset vibrotactile stimulation induces sustained cumulative benefits in Parkinson’s disease. *Front. Physiol,* **12,** 624317. 10.3389/fphys.2021.624317 (2021).

The original Article has been corrected.

